# Structure, Dynamics, and Wettability of Water at Metal Interfaces

**DOI:** 10.1038/s41598-019-51323-5

**Published:** 2019-10-15

**Authors:** Suji Gim, Kang Jin Cho, Hyung-Kyu Lim, Hyungjun Kim

**Affiliations:** 1 Graduate School of EEWS and Department of Chemistry, Korea Advanced Institute of Science and Technology (KAIST), Yuseong-gu, Daejeon 34141 Korea; 2 Division of Chemical Engineering and Bioengineering, Kangwon National University, Chuncheon, Gangwon-do 24341 Korea

**Keywords:** Structure of solids and liquids, Surfaces, interfaces and thin films, Computational chemistry

## Abstract

The water/metal interface often governs important chemophysical processes in various technologies. Therefore, from scientific and engineering perspectives, the detailed molecular-level elucidation of the water/metal interface is of high priority, but the related research is limited. In experiments, the surface-science techniques, which can provide full structural details of the surface, are not easy to directly apply to the interfacial systems under ambient conditions, and the well-defined facets cannot be entirely free from contamination at the contact with water. To answer long-standing debates regarding the wettability, structure, and dynamics of water at metal interfaces, we here develop reliable first-principles-based multiscale simulations. Using the state-of-the-art simulations, we find that the clean metal surfaces are actually superhydrophilic and yield zero contact angles. Furthermore, we disclose an inadequacy of widespread ice-like bilayer model of the water adlayers on metal surfaces from both averaged structural and dynamic points of view. Our findings on the nature of water on metal surfaces provide new molecular level perspectives on the tuning and design of water/metal interfaces that are at the heart of many energy applications.

## Introduction

The water/metal interface is ubiquitous in a variety of interesting systems that are relevant to heterogeneous catalysts, electrochemistry, corrosion, fluid transport, etc., which all have a great deal of technological importance in our daily lives. Notably, the proper control and optimization of such systems are prerequisites to resolving the current climate change and renewable energy issues. Therefore, there is an utmost scientific need to develop a fundamental understanding of water/metal interfaces. However, many chemophysical properties of water/metal interfaces are still unknown. For example, even one of the most basic properties, the water contact angle (*θ*_CA_), shows huge discrepancies among the various measurements at gold surfaces, spanning from 0° to 93° (Table S1). This discrepancy has raised the question of whether a clean gold surface is hydrophilic or hydrophobic^1^.

Of prime interest is the identification of the atomic/molecular structure of the interface. However, unlike a bare surface, the interface is unexposed and thereby difficult to directly observe. Furthermore, most experimental techniques that can reveal the atomic arrangements require ultrahigh-vacuum (UHV) conditions, but water is stable only at the cryogenic temperature under UHV conditions. This leads most experimental efforts to be focused on elucidating the structure of ice clusters on metal surfaces within cryostats^2^, while the room-temperature (RT) structure and dynamics of water at metal interfaces are largely unknown.

Due to the aforementioned experimental difficulties, molecular level investigations of the water/metal interfaces at RT seem to be, *per se*, suitable for theoretical studies^3^. However, a single simulation method can hardly describe the high complexity of the interfacial systems. For example, density functional theory (DFT) can accurately describe the electronic polarization of a metal surface, and the interaction potential between the water and metal surface relies on a proper choice of nonlocal correlation functionals^4^. However, the system size and timescale are quite limited, as only a few hundred atoms and picoseconds of measurement are affordable with regard to computational cost, thereby hampering the ability to obtain a full understanding of the liquid structure and dynamics. On the other hand, classical molecular dynamics (MD) simulations have been a powerful tool to examine liquid structures and dynamics, but accurate force-field (FF) parameters need to be established to ensure the reliability of the simulation results.

**Figure 1:**
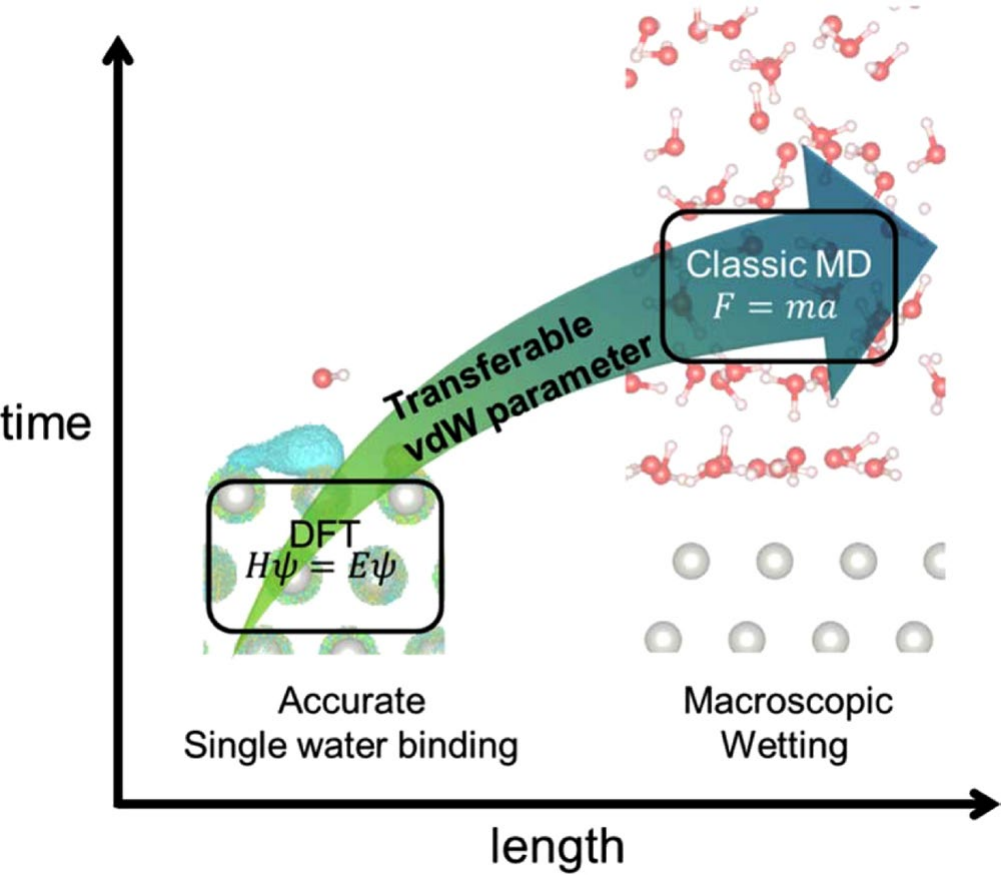
Schematic diagram of the first-principles-based multiscale simulation of a solid (metal)-liquid (water) interfacial system. The classical treatment of water molecules and the quantum description of the metal surface enable an accurate description of both the single water-metal surface interaction and the liquid water-metal interfacial interaction using a single set of transferrable vdW parameters.

Here, we investigate the water/metal interface by employing our recently developed first-principles-based multiscale simulation method (Fig. 1), which is called the density functional theory in classical explicit solvents (DFT-CES)^5–8^. Using the mean-field coupling of electrostatics between the DFT and classical molecular dynamics, DFT-CES efficiently describes both the structural and dynamical properties of liquid water and the full electronic details and surface polarization of the metal surface. In addition to the seamless treatment of the electrostatics, DFT-CES employs the parameterized pairwise interaction terms to account for the exchange repulsion and long-range nonlocal correlation (namely, dispersion) energies that are missing during the classical treatment of water. When these van der Waals (vdW) parameters are carefully prepared to reproduce the single water binding curve from the first-principles, our recent study has demonstrated that the DFT-CES can accurately describe the macroscopic wettability of the solid surface without having experimental input or empirical treatment^6^.

## Wettability of Clean Metal Surfaces

To properly account for the nonlocal correlation governing the water binding on the metal surface^4^, in this study, we employ the nonlocal correlation functional DFT, vdW-DF2^9^, which has often been used to investigate water structures on metal at zero kelvin^10^. Using vdW-DF2, we obtain a single water-metal binding reference curve for Ag, Au, Pd, and Pt, respectively (Fig. S1), and determine the corresponding vdW parameters (Table S2).

Using the DFT-CES calculations of the water/metal interfacial systems (Fig. S2), we calculate the surface wettability of the clean metal surfaces of Ag, Au, Pd, and Pt for the atomically flat facets of (111) and (100), as shown in Table 1. The surface wettability is quantified using the work of adhesion (*W*_ad_) that is related to the contact angle (*θ*_CA_) through the Young-Dupré equation *W*_ad_ = *γ*_lv_(1 + cos *θ*_CA_), where *γ*_lv_ is the surface tension of water. Despite the previous debate on the hydrophobicity of clean metal surfaces^1^, we find that all clean metal surfaces that are investigated here have zero *θ*_CA_, which is where water exhibits complete spreading-out behavior. This seems to be in agreement with the general consensus that noble metal surfaces are hydrophilic.

**Table 1:** Predicted work of adhesion (*W*_ad_) and water contact angle (*θ*_CA_) of different metal surfaces.

	(111)		(100)	
	*W*_ad_ (mJ/m^2^)	*θ*_CA_ (degrees)	*W*_ad_ (mJ/m^2^)	*θ*_CA_ (degrees)
Ag	195.81	0	179.00	0
Au	217.16	0	201.70	0
Pd	320.70	0	300.85	0
Pt	267.20	0	255.52	0

The vdW parameters of the DFT-CES simulations are optimized to reproduce the reference curves from the vdW-DF2 functional. Units are in mJ/m^2^ for *W*_ad_, and degrees for *θ*_CA_.

Since *θ*_CA_ becomes zero when *W*_ad_ is merely larger than 2*γ*_lv_ ≈ 145 mJ/m^2^, the experiment-theory comparison of *θ*_CA_ is less informative. Indeed, our predicted *W*_ad_ values are nearly one order of magnitude smaller than other previous theoretical ones that were obtained using classical force-fields^11^, albeit both results yield zero *θ*_CA_. We thus compare our values with the references that are derived from experimental dielectric spectroscopic data. Using Lifshitz theory^12–15^, one can calculate the Hamaker coefficient^16–19^ from spectroscopic data, which can be converted into *W*_ad_ assuming that the vdW interaction predominantly determines the water-metal interaction. For the Ag and Au surfaces, the *W*_ad_ values that are estimated using Lifshitz theory span 110–180 mJ/m^2^ and 110–200 mJ/m^2^, respectively. These are highly comparable with our values of 180–196 mJ/m^2^ and 202–217 mJ/m^2^ for Ag and Au facets, respectively. We emphasize that our first-principles-derived results provide a reliable theoretical measure of the wettability of ideally clean metal surfaces without incurring any uncontrollable experimental arbitrariness that is caused from surface oxidation, contaminants, etc. To assess the possible error due to the imperfect description of DFT functionals, we calculate the *W*_ad_ values using the vdW parameters that are obtained from another functional of vdW-DF2^c09x ^^20^ (Fig. S3 and Table S2), which leads to only small quantitative changes (Table S3), but we draw the same conclusion as was discussed above.

## Structure of Water Adlayer at Metal Interfaces

Based on the accurate description of the water/metal interface, we now understand the structure and dynamics of interfacial water. The local density profile of water along the surface normal direction (chosen as the z-direction) shows a layering tendency near the solid surface due to the symmetry breaking from the bulk, as widely shown from various solid-fluid interfaces^21,22^. Of particular, the local density shows two prominent peaks, implying the existence of strong adsorption water layers at the hydrophilic metal interface (Fig. S4). This is in consistent with the previous results from MD simulation using polarizable force fields^23^ and quantum mechanical MD simulation^24^, both of which report the existence of two prominent peaks in the local water density profile. Interestingly, a similar density profile is observed for the water at the hydrophobic surface such as graphene, graphite or fluorographene (Fig. S5a–c), while the air-water interface shows a completely different water density profile where the layering is lacking (Fig. S5d). We thus conclude that the water bilayer is formed at the interfacial region regardless of the surface hydrophilicity. We note that the “bilayer” term is used here in a loose sense, while it often refers to the buckled hexagonal water layer resembling the ice structure in the field^25^.

**Figure 2:**
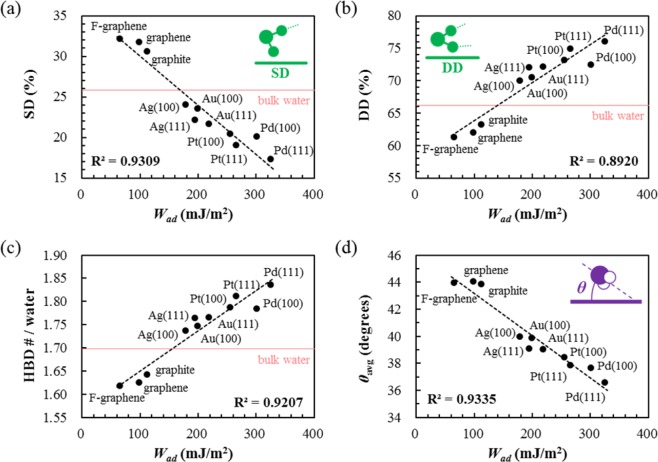
Populations of different hydrogen bond (HB) configurations of the water adlayers on metal surfaces as a function of the surface hydrophilicity (*W*_ad_) for (**a**) single donor (SD) configuration and (**b**) double donor (DD) configuration. (**c**) Number of hydrogen bond donors (HBDs) per water molecule in the adlayer region as a function of the surface hydrophilicity (*W*_ad_). (**d**) Average angle of the water dipole from the metal surface in the adlayer region as a function of the surface hydrophilicity (*W*_ad_).

We then characterize the structure of water in the first layer contacting the surface, namely, a water adlayer. The hydrogen bond (HB) configuration of the water adlayer gradually changes from the single-donor (SD) to double-donor (DD) type (Fig. 2a,b) with an increase of the *W*_ad_, which consequently accompanies an increase of the HB donor (HBD) number per water (Fig. 2c), i.e., strengthening the HB network. The existence of a strong linear correlation between the SD/DD population and the surface hydrophilicity (*W*_ad_) infers that the microscopic HB configuration is a good descriptor of the macroscopic surface wetting property.

The enhanced HB network at the hydrophilic surface is seemingly opposed to the simple conceptual picture that the strong attraction between the water and solid surface would competitively weaken the HB interaction among water molecules. We further elucidate that such a rather counterintuitive behavior becomes possible since the stronger attraction to the surface yields the more lying-down orientation of water (Fig. 2d) by stabilizing the lone pairs of water molecules^26^. This geometrically enables greater hydrogen bonds (HB) among interfacial water molecules.

When *W*_ad_ becomes larger than 155 mJ/m^2^ (Fig. 2c), as for most metal surfaces, the number of HBDs becomes larger than that of bulk liquid water (1.70), and may even be close to 2, which is the value of the hexagonal ice structure. It is thus natural to question whether the water adlayer structure becomes actually hexagonal ice-like.

## Topology of Hydrogen Bond Network at Interfaces

**Figure 3:**
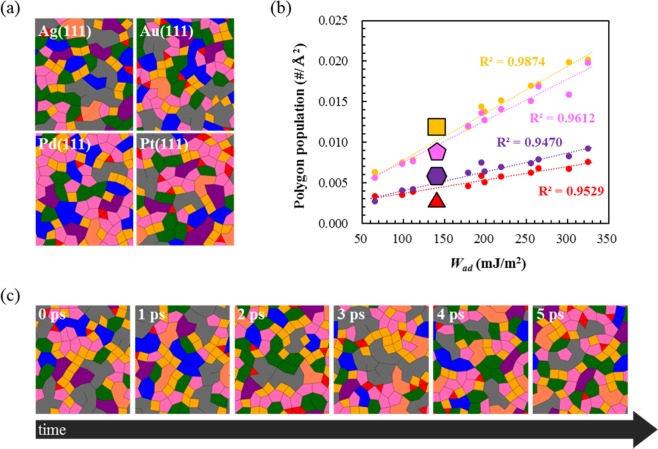
(**a**) Topology of the hydrogen bond (HB) network of water in the adlayer region on Ag(111), Au(111), Pd(111), and Pt(111) surfaces. Representative cases are shown. (**b**) Population of the small polygons (*n*-gon) that are formed by enclosures of HB, where *n* = 3 (red), 4 (yellow), 5 (pink), and 6 (purple), as a function of the surface hydrophilicity (*W*_ad_). (**c**) Temporal series of HB topological changes that are displayed at every picosecond. The Pd(111) case is representatively shown.

To answer if the interfacial water is in the ice-like phase, we analyze the topology of the HB network of the water adlayer using HB connectivity (as defined in the Fig. S6) as shown in Fig. 3a, and then investigate the population of small polygons (*n*-gon: *n* = 3, 4, 5, and 6) at different interfaces. We find that the more polygons are formed at the more hydrophilic surface due to the increased number of HBDs (Fig. 3b). However, regardless of the surface hydrophilicity, tetragons and pentagons are more populated than hexagons. From the (averaged) structural point of view, therefore, the water adlayer is not quite likely to be “hexagonal” ice-like.

More interesting is the dynamics of the water adlayer. As representatively shown in the snapshots for the water at the Pd(111)/water interface (Fig. 3c), which are taken at every picosecond, the HB topology shows a rapid change (<1 ps). Thus, also from the dynamics point of view, it is difficult to conclude that the HB network of the water adlayer is either solid or “ice”-like.

## Interfacial Water Shows Liquid-Phase Dynamics

**Figure 4:**
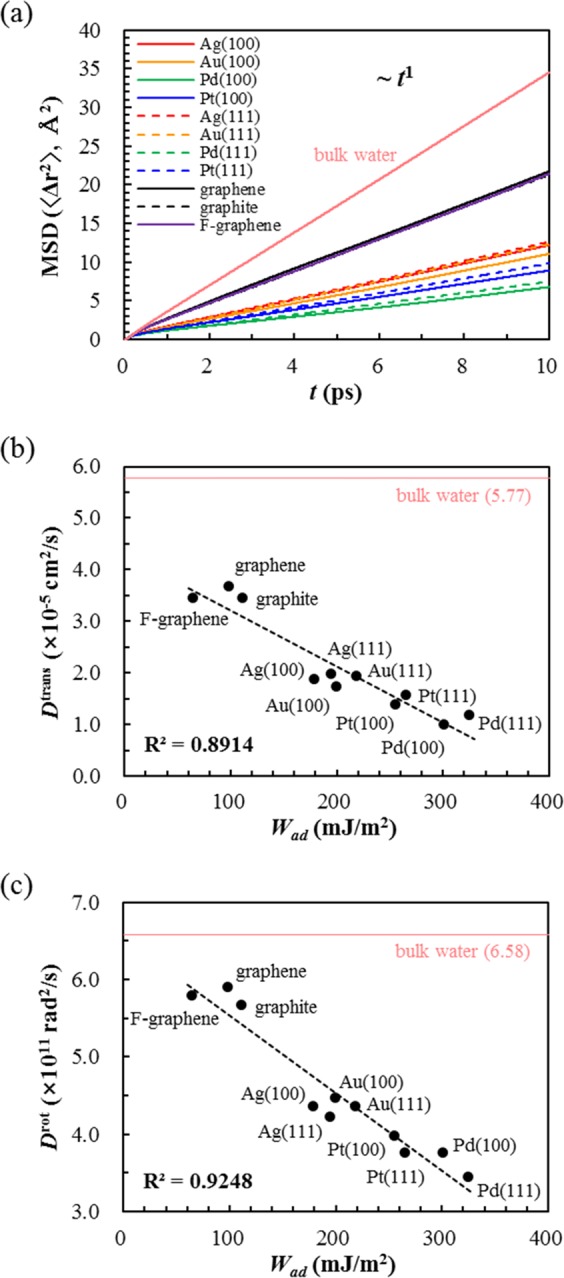
(**a**) Mean-squared displacement (MSD) of the water molecules that started their diffusion from the adlayer region. For comparison, the MSD of bulk water is shown in the graph. (**b**) Translational (*D*^trans^) and (**c**) rotational (*D*^rot^) diffusion coefficients of water at the adlayer region are shown as a function of the surface hydrophilicity (*W*_ad_). For comparison, the *D*^trans^ and *D*^rot^ of bulk water are shown as horizontal lines.

From the mean-squared displacement (MSD) of the water molecules that started their diffusion from the adlayer region (Fig. 4a), we further elucidate that the diffusion of the water adlayer follows normal Fickian behavior, thereby supporting the nonexistence of the ice-like phase. Considering that the flattening-out behavior of MSD in the log-log scale is a key fingerprint of supercooled water^27^, the absence of such a feature in our case (Fig. S7) further excludes the possibility of a glassy phase at the interface, and thus the phase of the water adlayer is concluded to be a simple liquid.

Compared with bulk liquid water, however, the diffusivity of the interfacial liquid water shows a quantitative difference. Figure 4b,c show that the translational (*D*^trans^) and rotational diffusion constants (*D*^rot^.) gradually decrease as the surface hydrophilicity increases (values are list in Table S4). We note that *D*^trans^ and *D*^rot^ are obtained using the Green-Kubo relation, which yields the same value of the translational diffusion constant as the one that is obtained from the MSD using the Einstein-Enskog relation (Fig. S8). Similar to the *D*^trans^ of the water adlayer, which is smaller than that of bulk water in all cases, the *D*^rot^ of the water adlayer is also smaller than that of bulk water in all cases. In consideration of both *D*^trans^ and *D*^rot^, the liquid phase of the water adlayer diffuses slower than the bulk liquid water regardless of the surface hydrophilicity. Thus, our results clearly show no existence of hexagonal ice-like phase at the interface at ambient condition, instead a simple liquid phase exists with decreased diffusivity.

To conclude, using state-of-the art multiscale simulation, we elucidate that the clean noble metal surfaces are hydrophilic; therefore, water tends to spread out flat on them (*θ*_CA_ = 0). Our results agree well with the values that are calculated from the experimental spectroscopic data, thus suggesting that several previous exrimental reports of large contact angle values could be due to partial surface oxidation and/or contamination. Based on the accurate first-principles of the DFT description of water metal interaction, we further investigate the conventional picture of the water structure at the interface, e.g., an ice-like bilayer model. From both the structural and dynamics points of view, we conclude that there is an inadequacy hexagonal ice-like phase, and the interfacial water is in the liquid phase with retarded diffusivity. Our molecular-level understanding of water at metal interfaces provides a fundamental starting point to tackle important challenges in various technological processes that are related to water/metal interfaces, e.g., fouling inhibition, anti-icing surfaces, electrical double layers formation, etc.

## Methods

### Single-scale density functional theory (DFT) calculations

To obtain single water binding reference curves, we performed DFT calculations using Quantum ESPRESSO^28^ by employing two different non-local correlation functionals of vdW-DF2^9^ and vdW-DF2^c09x^^20^. The metal slab was modeled using 3 layers of (2 × 2) surface unit cell of (111) surface (consisting of 48 metal atoms), and the electronic-ion interactions were considered in the form of the projector-augmented-wave (PAW) method^29^. The kinetic energy cutoff for the planewaves was set as 50 Ry, and the Gaussian smearing was used with a value of 0.2 eV for Brillouin-zone integration in metals. The dipole correction was applied along the surface normal direction (chose as a *z*-direction).

### Multi-scale simulations: DFT in classical explicit solvents (DFT-CES)

The DFT-CES method is implemented by combining open-source density functional theory (DFT) and classical molecular dynamics (MD) programs; Quantum ESPRESSO^28^ (a planewave DFT code) and Large-scale Atomic/Molecular Massively Parallel Simulator^30^ (LAMMPS; a classical MD code). Detailed simulation procedure of DFT-CES can be found from our previous publications^5,6^.

DFT part was described using Perdew-Burke-Ernzerhof (PBE) exchange-correlation functional^31^. The metal slab was modeled using 3 layers of (2 × 2) surface unit cell of (111) surface (consisting of 48 metal atoms), and 3 layers of (2 × 2) surface unit cell of (100) surface (consisting of 24 metal atoms), and the electronic-ion interactions were considered in the form of the projector-augmented-wave (PAW) method. The kinetic energy cutoff for the planewaves was set as 50 Ry, and the Gaussian smearing was used with a value of 0.2 eV for Brillouin-zone integration in metals. The dipole correction was applied along the surface normal direction (chose as a *z*-direction).

For MD simulations, we used the (3 × 3) and (4 × 4) expanded supercell structure of aforementioned (111) and (100) slab models, respectively, in order to minimize the finite size effect during MD simulations. We simulated 1,000 number of TIP3P-Ew water molecules^32^ at the metal interfaces. By employing the Nosé-Hoover thermostat, canonical ensemble (NVT) simulation was performed at 300 K for 1 ns at every DFT-CES iteration, and the last 500 ps trajectory was used to compute the ensemble averaged solvent charge density. The long-range electrostatic interactions were calculated using the multi-level summation method (MSM)^33^. The DFT-CES iteration was performed until the internal energy change of the DFT part became less than 0.1 kcal/mol. Optimized vdW parameters for interfacial interaction can be found from; the reference^6^ for C of graphene/graphite systems; Fig. S9 for F of fluorographene (F-graphene); and Table S2 for metals.

## Supplementary information

Supplementary information
