# Supplementary material for: Structure, Dynamics, and Wettability of Water at Metal Interfaces
Source: Sci Rep. 2019 Oct 15;9:14805. doi: 10.1038/s41598-019-51323-5 (PMC6794260; doi:10.1038/s41598-019-51323-5)
Supplement: Supplementary file 1 — Supplementary information [file 41598_2019_51323_MOESM1_ESM.docx]

Supporting information for:

**Structure, Dynamics, and Wettability of Water at Metal Interfaces**

Suji Gim^†,∥^, Kang Jin Cho^†,∥^, Hyung-Kyu Lim^*,‡^, and Hyungjun Kim^*,†^

^†^Graduate School of EEWS and Department of Chemistry, Korea Advanced Institute of Science and Technology (KAIST), Yuseong-gu, Daejeon 34141, Korea

^‡^Division of Chemical Engineering and Bioengineering, Kangwon National University, Chuncheon, Gangwon-do 24341, Korea

^*^Correspondence to H.-K. L. ([hklim@kangwon.ac.kr](mailto:hklim@kangwon.ac.kr)) and H.K. ([linus16@kaist.ac.kr](mailto:linus16@kaist.ac.kr))


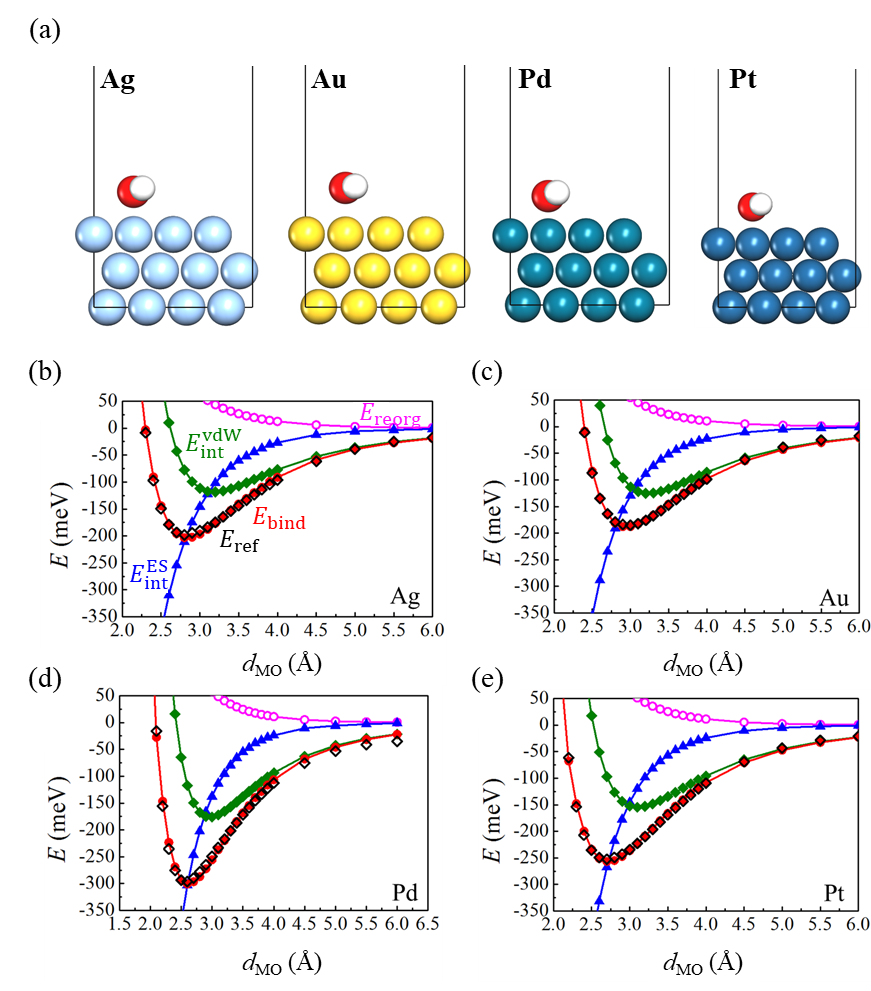


**Figure S1.** (a) DFT simulation cell for calculating the reference curve of the single water binding energy as a function of the metal-water distance ($d_{\mathrm{MO}}$) on metal (111) surfaces using the vdW-DF2 functional. The metal slab consists of 16 atoms in a periodic boundary cell. The reference binding curves for (b) Ag, (c) Au, (d) Pd, and (e) Pt are shown in black ($E_{\mathrm{ref}}$), which are reproduced using the DFT-CES binding energy ($E_{\mathrm{bind}}$), as shown in red. The DFT-CES energetics consist of the reorganization energy due to the electron polarization of the metal ($E_{\mathrm{reorg}}$), as shown in magenta; the electrostatic interaction energy between the water dipole and polarized electron cloud ($E_{\mathrm{int}}^{\mathrm{ES}}$), as shown in blue; and the parameterized vdW interaction energy between the water and metal surface ($E_{\mathrm{int}}^{\mathrm{vdW}}$), as shown in green. (i.e., $E_{\mathrm{bind}}=E_{\mathrm{reorg}}+E_{\mathrm{int}}^{\mathrm{ES}}+E_{\mathrm{int}}^{\mathrm{vdW}}$) $E_{\mathrm{int}}^{\mathrm{vdW}}$, which is modeled using the pairwise Buckingham potential ($E_{\mathrm{int}}^{\mathrm{vdW}}=\sum_{i<j} E_{exp-6}\left( r_{ij} \right)$, where $E_{exp-6}\left( r_{ij} \right)=\varepsilon_{0,ij}\left[ 6/\left( \alpha_{ij}-6 \right)\exp\left\{ \alpha_{ij}\left( 1-r_{ij}/r_{0,ij} \right) \right\}-\alpha_{ij}/\left( \alpha_{ij}-6 \right)\left( r_{0,ij}/r_{ij} \right)^{6} \right]$) that is used to describe the Pauli repulsion of the diffuse electron density of metal. The fitted vdW parameters are summarized in Table S2.


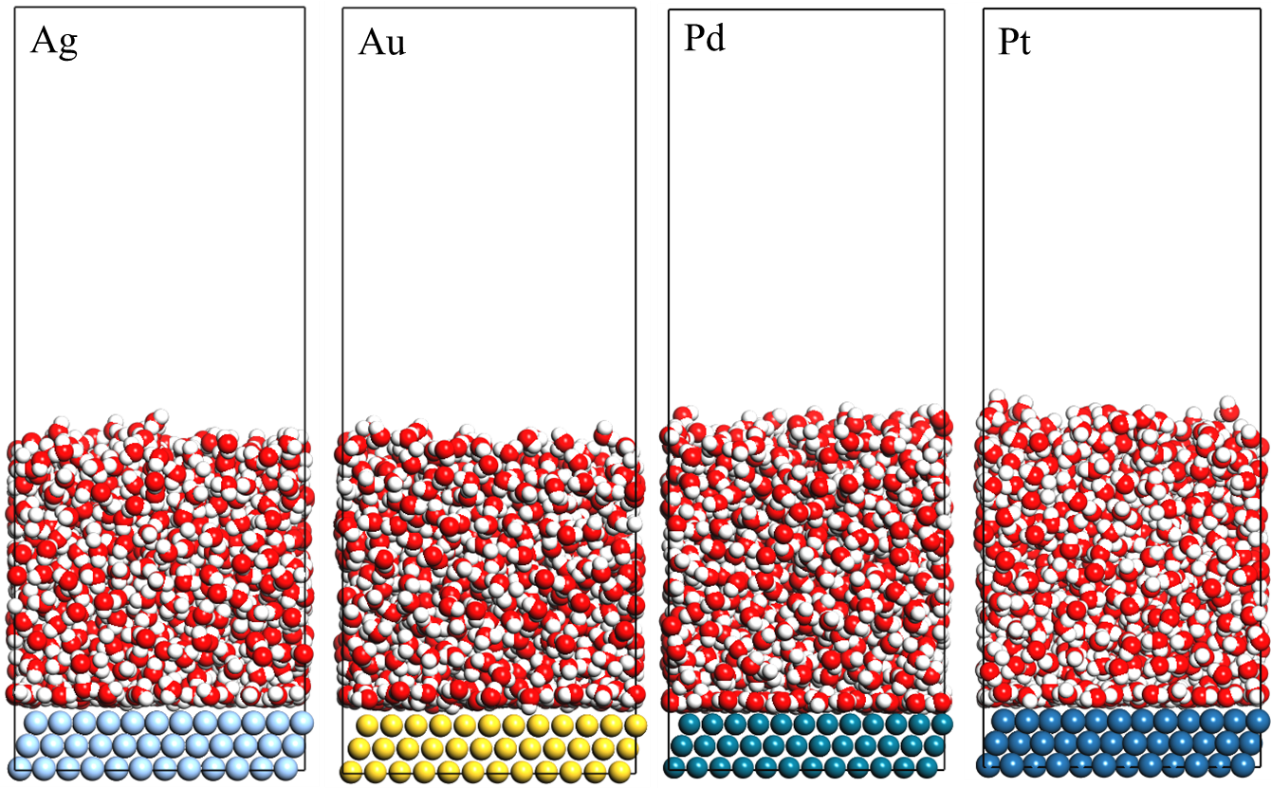


**Figure S2.** DFT-CES simulation cells of water/metal interfacial systems. All representative snapshots were taken from (111) surfaces.


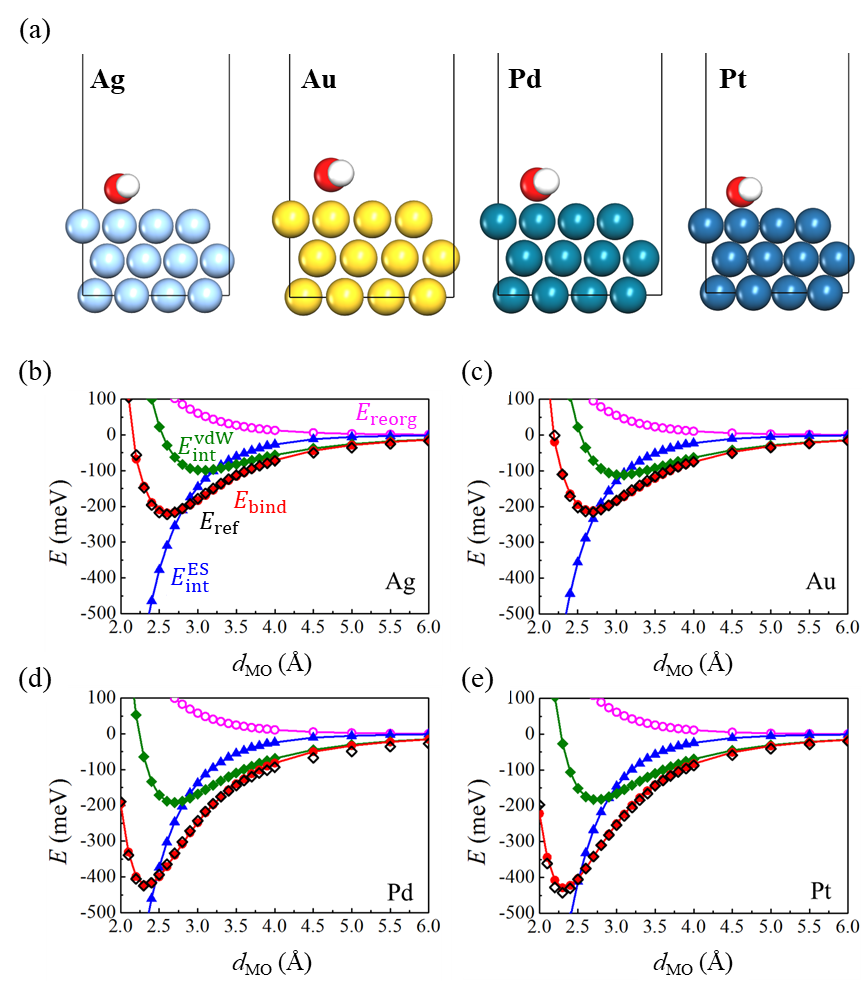


**Figure S3.** (a) DFT simulation cell for calculating the reference curve of the single water binding energy as a function of the metal-water distance ($d_{\mathrm{MO}}$) on metal (111) surfaces using the vdW-DF2^c09x^ functional. The metal slab consists of 16 atoms in a periodic boundary cell. The reference binding curves for (b) Ag, (c) Au, (d) Pd, and (e) Pt are shown in black ($E_{\mathrm{ref}}$), which are reproduced using the DFT-CES binding energy ($E_{\mathrm{bind}}$), as shown in red. The DFT-CES energetics consist of the reorganization energy due to the electron polarization of the metal ($E_{\mathrm{reorg}}$), as shown in magenta; the electrostatic interaction energy between the water dipole and polarized electron cloud ($E_{\mathrm{int}}^{\mathrm{ES}}$), as shown in blue; and the parameterized vdW interaction energy between the water and metal surface ($E_{\mathrm{int}}^{\mathrm{vdW}}$), as shown in green. (i.e., $E_{\mathrm{bind}}=E_{\mathrm{reorg}}+E_{\mathrm{int}}^{\mathrm{ES}}+E_{\mathrm{int}}^{\mathrm{vdW}}$) $E_{\mathrm{int}}^{\mathrm{vdW}}$, which is modeled using the pairwise Buckingham potential ($E_{\mathrm{int}}^{\mathrm{vdW}}=\sum_{i<j} E_{exp-6}\left( r_{ij} \right)$, where $E_{exp-6}\left( r_{ij} \right)=\varepsilon_{0,ij}\left[ 6/\left( \alpha_{ij}-6 \right)\exp\left\{ \alpha_{ij}\left( 1-r_{ij}/r_{0,ij} \right) \right\}-\alpha_{ij}/\left( \alpha_{ij}-6 \right)\left( r_{0,ij}/r_{ij} \right)^{6} \right]$) that is used to describe the Pauli repulsion of the diffuse electron density of metal. The fitted vdW parameters are summarized in Table S2.


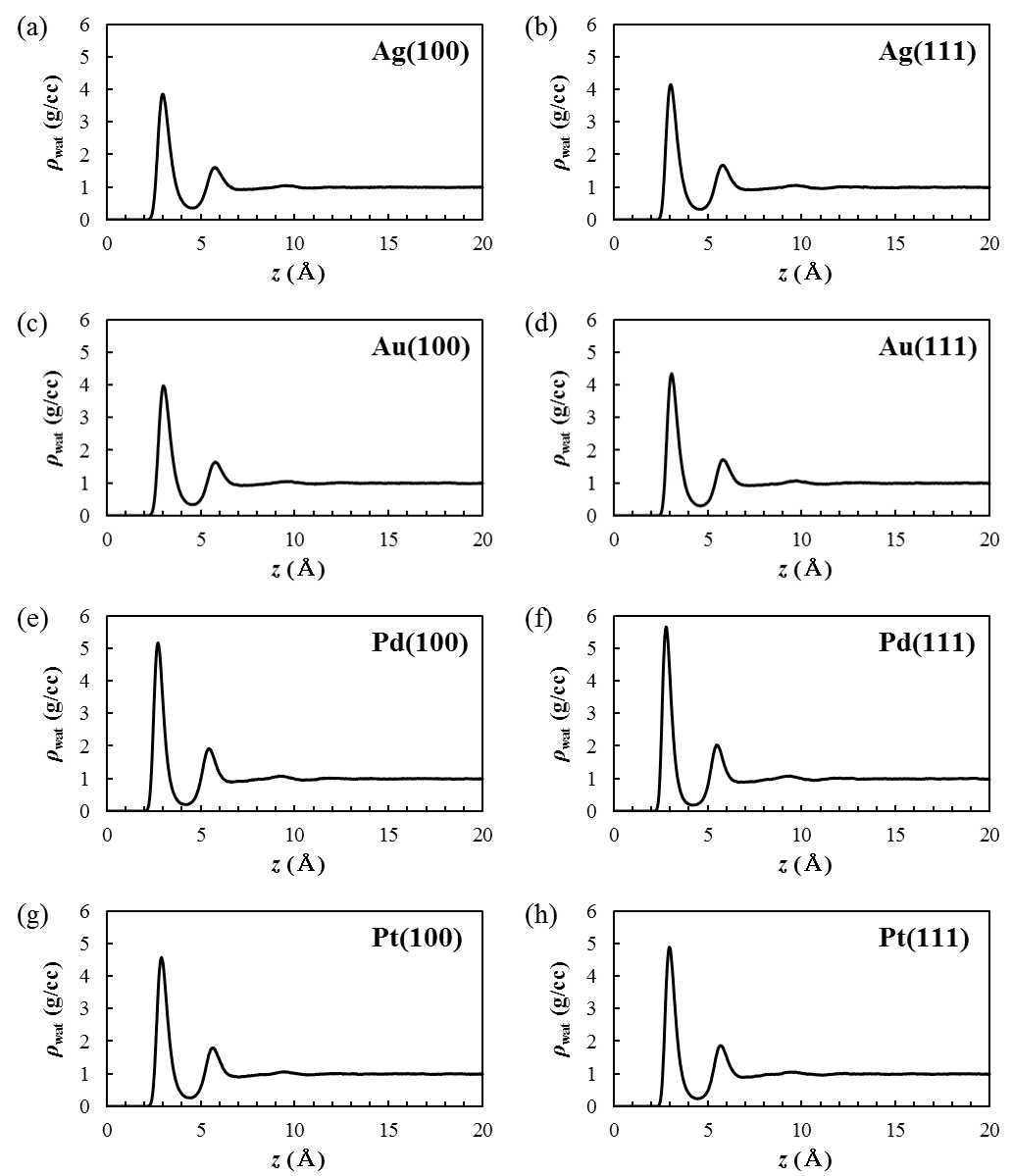


**Figure S4.** Local density profile of water molecules ($\rho_{\mathrm{wat}}$) along the surface normal direction (chosen as z-direction) for (a) Ag(100), (b) Ag(111), (c) Au(100), (d) Au(111), (e) Pd(100), (f) Pd(111), (g) Pt(100), and (h) Pt(111) surfaces.


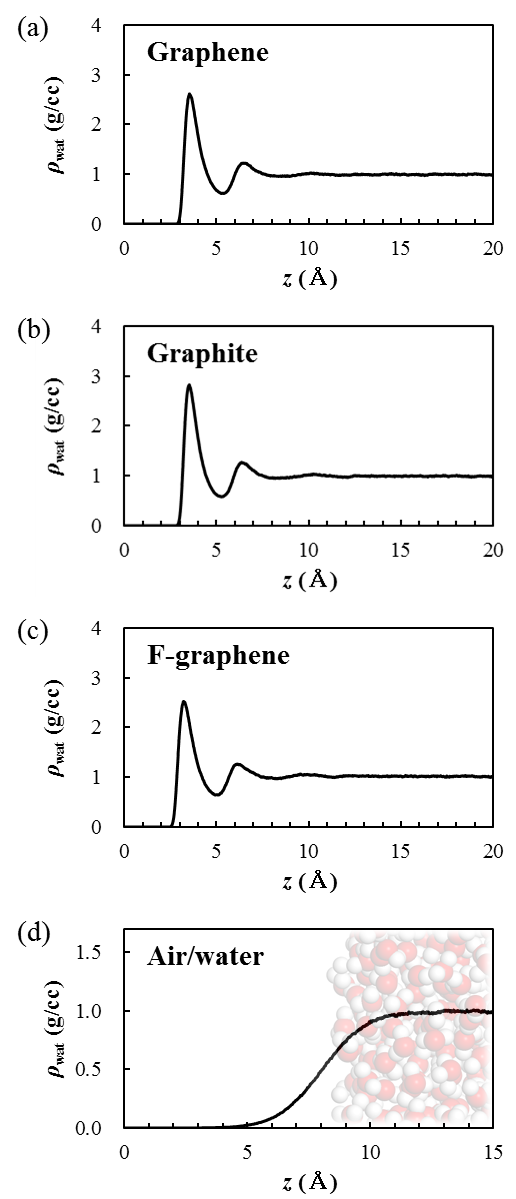


**Figure S5.** Local density profile of water molecules ($\rho_{\mathrm{wat}}$) along the surface normal direction (chosen as z-direction) for (a) graphene, (b) graphite, and (c) fluorographene (F-graphene), compared with the $\rho_{\mathrm{wat}}$ at (d) the air/water interface.


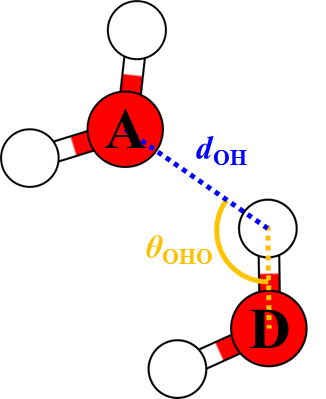


**Figure S6.** Geometrical definition of the hydrogen bond (HB) that is used in this paper, which reproduces a HB number of 3.5 for bulk water (TIP3P-Ew model). The A and D marks on oxygen atoms denote the HB acceptor and donor, respectively. A HB is defined as when the distance between oxygen atom at acceptor and hydrogen atom at donor ($d_{\mathrm{OH}}$) is shorter than 2.5$Å$ and the angle $\angle O_{A}HO_{D}$ ($\theta_{\mathrm{OHO}}$) is smaller than 120º.


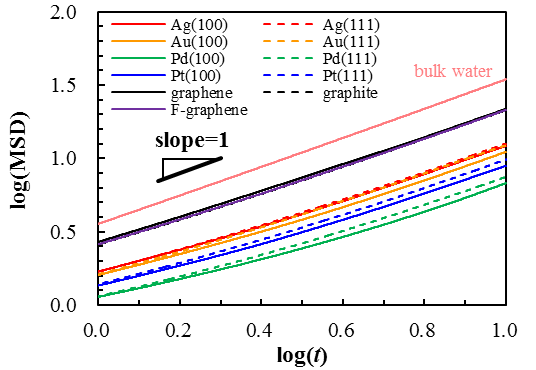


**Figure S7.** Mean-squared displacement (MSD) of the water molecules that started their diffusion from the adlayer region with log-log plot. For comparison, the MSD of bulk water is shown in the graph. The slopes of MSD in all cases with log-log plot are close to unity.


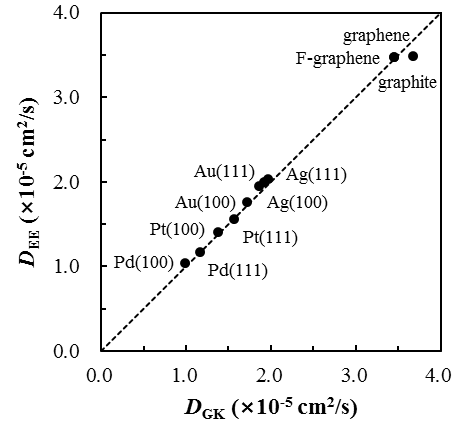


**Figure S8.** Correspondence between the diffusion coefficient values from the Green-Kubo relation ($D_{\mathrm{GK}}$) and those from the Einstein-Enskog relation ($D_{\mathrm{EE}}$).


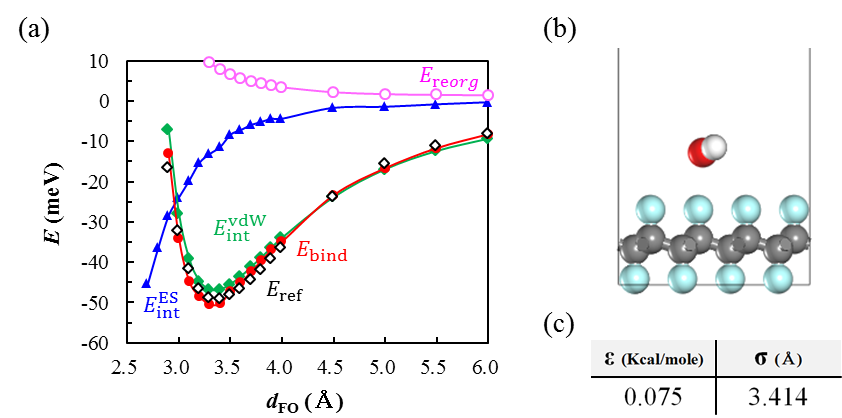


**Figure S9.** (a) The binding energy curves as a function of the distance ($d_{\mathrm{FO}}$) between a fluorographene (F-graphene) surface and a water using the vdW-DF2^c09x^ functional. The reference binding curve is shown in black ($E_{\mathrm{ref}}$), which is reproduced using the DFT-CES binding energy ($E_{\mathrm{bind}}$), as shown in red. The DFT-CES energetics consist of the reorganization energy due to the electron polarization of the slab ($E_{\mathrm{reorg}}$), as shown in magenta; the electrostatic interaction energy between the water dipole and polarized electron cloud ($E_{\mathrm{int}}^{\mathrm{ES}}$), as shown in blue; and the parameterized vdW interaction energy between the water and slab ($E_{\mathrm{int}}^{\mathrm{vdW}}$), as shown in green. (i.e., $E_{\mathrm{bind}}=E_{\mathrm{reorg}}+E_{\mathrm{int}}^{\mathrm{ES}}+E_{\mathrm{int}}^{\mathrm{vdW}}$) $E_{\mathrm{int}}^{\mathrm{vdW}}$ is modeled using the 12-6 Lennard-Jones potential. (b) DFT simulation cell for calculating the reference curve of the single water binding energy. (c) The fitted vdW parameters for the Lennard-Jones potential.

**Table S1.** Contact angles of gold surfaces from various experimental reports.

| $\boldsymbol{\theta}_{\mathbf{CA}}$ **(º)** | **Year** | **Reference** |
| --- | --- | --- |
| 7 | 1953 | J. Phys. Chem., 57, 1965 |
| 64-68 | 1954 | Faraday Discuss., 18, 74 |
| 0 | 1955 | J. Phys. Chem., 59, 1097 |
| > 60 | 1964 | Ind. Eng. Chem. Res., 56, (12), 40 |
| 65 | 1964 | J. Phys. Chem., 68, 3038 |
| 0 | 1964 | J. Phys. Chem., 68, 1804 |
| 0 | 1965 | J. Phys. Chem., 69, 4238 |
| 62 | 1968 | J. Phys. Chem., 72, 2412 |
| 0 | 1970 | J. Phys. Chem., 74, 2313 |
| 0 | 1970 | J. Phys. Chem., 74, 2309 |
| 0 | 1970 | J. Phys. Chem., 74, 2313 |
| 50-70 | 1971 | J. Colloid Interface Sci., 37, 410 |
| 0 | 1972 | J. Electroanal. Chem., 44, 51 |
| 50-79 | 1975 | J. Electroanal. Chem., 62, 313 |
| 0 | 1977 | J. Electroanal. Chem., 81, 285 |
| 0 | 1980 | J. Colloid Interface Sci., 75, 1 |
| 77.4 | 2012 | Nat. Mater., 11, 217 |
| 69 | 2017 | ACTA Phys. Pol. A., 132, (1), 185 |

**Table S2.** Buckingham potential parameters for the metal (M)-oxygen (O) of water. Units are kcal/mol for $\varepsilon_{0,MO}$, $Å$ for $r_{0,MO}$, and $\alpha_{\mathrm{MO}}$ is dimensionless.

| Reference  method |  | Ag | Au | Pd | Pt |
| --- | --- | --- | --- | --- | --- |
| vdW-DF2 | $\varepsilon_{0,MO}$ | 0.4308 | 0.4439 | 0.6525 | 0.5083 |
|  | $r_{0,MO}$ | 3.5785 | 3.6493 | 3.3450 | 3.5325 |
|  | $\alpha_{\mathrm{MO}}$ | 10.9135 | 10.9607 | 10.5424 | 10.5569 |
| vdW-DF2^c09x^ | $\varepsilon_{0,MO}$ | 0.4194 | 0.4829 | 1.0629 | 0.9964 |
|  | $r_{0,MO}$ | 3.4023 | 3.4005 | 2.9292 | 2.9791 |
|  | $\alpha_{\mathrm{MO}}$ | 11.0641 | 11.0574 | 10.9638 | 10.9922 |

**Table S3.** Predicted work of adhesion ($W_{\mathrm{ad}}$) and water contact angle ($\theta_{\mathrm{CA}}$) of different metal surfaces. The vdW parameters of the DFT-CES simulations are optimized to reproduce the reference curves from the vdW-DF2^c09x^ functional. Units are in mJ/m^2^ for $W_{\mathrm{ad}}$, and degrees for $\theta_{\mathrm{CA}}$.

|  | (111) | | (100) | |
| --- | --- | --- | --- | --- |
|  | $W_{\mathrm{ad}}$ (mJ/m^2^) | $\theta_{\mathrm{CA}}$ (degrees) | $W_{\mathrm{ad}}$ (mJ/m^2^) | $\theta_{\mathrm{CA}}$ (degrees) |
| Ag | 151.80 | 0 | 137.80 | 0 |
| Au | 180.73 | 0 | 164.75 | 0 |
| Pd | 446.25 | 0 | 452.51 | 0 |
| Pt | 221.58 | 0 | 202.60 | 0 |

**Table S4.** Translational and rotational diffusion coefficients ($D^{\mathrm{trans}}$ and $D^{\mathrm{rot}}$, respectively) of bulk and interfacial water molecules.

|  | $\boldsymbol{D}^{\mathbf{trans}}$  $\left( \times{10}^{-5} \mathrm{cm}^{2}/s \right)$ | $\boldsymbol{D}^{\mathbf{rot}}$  $\left( \times{10}^{11} \mathrm{rad}^{2}/s \right)$ |
| --- | --- | --- |
| Bulk | 5.768 | 6.584 |
| Ag(100) | 1.869 | 4.354 |
| Au(100) | 1.724 | 4.463 |
| Pd(100) | 0.993 | 3.759 |
| Pt(100) | 1.384 | 3.970 |
| Ag(111) | 1.976 | 4.225 |
| Au(111) | 1.927 | 4.352 |
| Pd(111) | 1.176 | 3.438 |
| Pt(111) | 1.572 | 3.752 |
| graphene | 3.676 | 5.894 |
| graphite | 3.455 | 5.665 |
| F-graphene | 3.454 | 5.792 |
